# Intrinsic and Synthetic Stable Isotope Marking of Tsetse Flies

**DOI:** 10.1673/031.011.7901

**Published:** 2011-07-01

**Authors:** Rebecca Hood-Nowotny, Margarete Watzka, Leo Mayr, Solomon Mekonnen, Berisha Kapitano, Andrew Parker

**Affiliations:** ^1^ Insect Pest Control Laboratory, Joint FAO/IAEA Division of Nuclear Techniques in Food and Agriculture, International Atomic Energy Agency, Vienna, Austria; ^2^ Department of Chemical Ecology and Ecosystem Research, Vienna Ecology Centre, Faculty of Life Sciences University of Vienna, Althanstrasse 14 ,A-1090 Vienna, Austria; ^3^ Kaliti Tsetse Mass Rearing and Irradiation Centre, Southern Tsetse Fly Eradication Project (STEP), Ministry of Science and Technology, Akaki Kaliti Kefle Ketema W.27 K. 10, Debrezeyet Road, P.O. Box 7794, Addis Ababa, Ethiopia; ^4^ Southern Tsetse Eradication Project, Southern Regional Government Agricultural Bureau, P.O. Box 80, Awassa, Ethiopia; ^§^ Current address: Southern Tsetse Eradication Project, Ministry of Science and Technology, , P.O. Box 474, Hawassa, Ethiopia

**Keywords:** *Glossina pallidipes*, Glossinidae, mass rearing, SIT, sterile insect technique

## Abstract

The sterile insect technique has been successfully used to eliminate tsetse populations in a number of programs. Program monitoring in the field relies on the ability to accurately differentiate released sterile insects from wild insects so that estimates can be made of the ratio of sterile males to wild males. Typically, released flies are marked with a dye, which is not always reliable. The difference in isotopic signatures between wild and factory-reared populations could be a reliable and intrinsic secondary marker to complement existing marking methods. Isotopic signatures are natural differences in stable isotope composition of organisms due to discrimination against the heavier isotopes during some biological processes. As the isotopic signature of an organism is mainly dependent on what it eats; by feeding factory-reared flies isotopically different diets to those of the wild population it is possible to intrinsically mark the flies. To test this approach unlabeled samples of *Glossina pallidipes* (Austen) (Diptera: Glossinidae) from a mass rearing facility and wild populations were analyzed to determine whether there were any natural differences in signatures that could be used as markers. In addition experiments were conducted in which the blood diet was supplemented with isotopically enriched compounds and the persistence of the marker in the offspring determined. There were distinct natural isotopic differences between factory reared and wild tsetse populations that could be reliably used as population markers. It was also possible to rear artificially isotopically labeled flies using simple technology and these flies were clearly distinguishable from wild populations with greater than 95% certainty after 85 days of “release”. These techniques could be readily adopted for use in SIT programs as complimentary marking techniques.

## Introduction

Tsetse flies (*Glossina* spp., Diptera: Glossinidae) and the diseases they transmit continue to create significant barriers to economic development in Sub-Saharan Africa, by affecting human health through sleeping sickness, limiting livestock production, and economic exploitation due to trypanosomosis. The very high vectorial efficiency of tsetse means that the population must be controlled to a very low level or completely eliminated, which can best be achieved with area-wide integrated pest management (AW-IPM) (Hendrichs et al. 2007).

A powerful “final push” tool in AW-IPM is the sterile insect technique (SIT). In SIT natural populations are continually swamped with sterile insects, usually males, resulting in population crashes and ultimately elimination (Knipling 1979). In 1997 tsetse flies were eliminated from Zanzibar using AW-IPM and SIT was a central component of the elimination program.

Elimination programs, by definition, are only successful when the very last target pest is gone. SIT uses the sterile insects to seek out the last wild target pest, but for efficient monitoring the sterile insects must be clearly marked or distinguishable from the wild target in a way that does not affect its ability to find, win, and mate the wild target. A marking method which enables monitoring of sterile to wild insect ratios during initial stages of operations is also useful for planning and logistics. In initial stages of elimination when there are vast numbers of mass reared sterile and wild flies, occasional misclassification of wild or sterile flies will have little impact on the overall operation. However at the final stages of the elimination it is imperative to know whether a fly is sterile or wild, as misclassification of sterile insects as wild in the closing stages of an elimination program can lead to the expensive extension of unnecessary control operations.

It is clear that a marking method is required that can unequivocally distinguish mass reared sterile flies from wild flies especially at later stages of an elimination program. There are many means of marking insects (Hagler and Jackson 2001; Parker 2005) that vary in their efficiency and side—effects. Currently insects are mostly marked with oil—based dyes (Hendricks and Graham 1970; Graham and Mangum 1971; Hendricks 1971) or fluorescent dye (Enkerlin et al. 1996; FAO/IAEA/USDA 2003). Oil—based dyes have the advantage of being incorporated into the tissues of the insect, but can have detrimental effects (Schroeder et al. 1974). Fluorescent dyes are applied to fly pupae and dye transfers to the ptilinum of the adult as it emerges (Vreysen et al. 1999) and lodges in the sutures, and can be detected in the field using a UV light. However, external dye may be washed off by rain or wild flies may be contaminated with the dye in the traps and these dyes have also been reported to have adverse effects on behavior (Logan and Proverbs 1975). One way of overcoming these problems is to use intrinsic or synthetic stable isotope markers that are integrated into the tissues of the fly; these are not visible and have no physiological effect (Helinski et al. 2007).

For those unfamiliar with stable isotope technology, stable isotopes occur naturally in the environment. They are safe, not radioactive, and do not decay. These qualities make them useful natural tracers. An isotope of an element has the same atomic number as the element, but a different number of neutrons and thus a different atomic weight, which can be detected using isotope ratio mass spectrometry. Usually the stable isotope mass spectrometer is coupled to an automated elemental analyzer, so a sample size of only 10µg N and 150µg C is required for analysis, taking about ten minutes per analysis (Fry 2006; Hood-Nowotny and Knols 2007; Michener and Lajtha 2007; IAEA 2009). Natural isotopic signatures are standardized to an internationally accepted scale, due to the large differences in the abundances of the carbon and nitrogen isotopes (^12^C∼1.1%, ^13^C∼98.9%, and ^15^N∼0.3663%, ^14^N∼99.63%), ^13^C/^12^C and ^15^N/^14^N ratios are generally expressed in the delta notation in parts per thousand (‰) relative to the internationally recognized standards Vienna Pee Dee Belemnite (VPDB) (Gröning 2004) and atmospheric nitrogen, respectively. Enriched samples are usually expressed as atom % abundance of the total element, in some cases they are expressed as excess values, i.e. the enriched abundance minus the natural abundance in the environment.

Stable isotopes can be used in two ways, either, by adding substances enriched in the stable isotope in question and following their fate; or they can be used by studying the natural variations in isotopic signatures in the environment, which arise from discrimination for or against the heavier isotope in certain biochemical or physical pathways. This second method is termed natural abundance studies. Using natural abundance methods it is possible to trace food-web structure, migration patterns, feeding preferences, etc. (Hobson and Clark 1992; Ostrom et al. 1997; Wassenaar and Hobson 1998; Fantle et al. 1999; Hood-Nowotny and Knols 2007).

Using enrichment techniques overcomes some of the constraints of natural abundance techniques and allows a wide range of physiological and ecological parameters to be studied. There is a wide variety of commercially available stable isotopeenriched compounds available that have higher concentrations of the rarer isotope than the natural background (or natural abundance). These can be easily integrated into feeding regimes of target insects and can be used, for example, in capture—recapture (Caudill 2003), feeding preference (Benedict et al. 2009), and resource allocation studies (Haq et al. 2010). Intrinsic isotopic marking is based on the inherent isotopic differences in diets of the mass reared compared to wild insects. The isotopic signature of an insect is normally within one or two parts per thousand of the diet on which they have fed. The inherent isotopic differences in the diet are the result of the slight discrimination for or against the heavier isotopes during some biological, chemical, or kinetic processes. Tsetse flies are adenoviviparous, meaning they produce one fully formed larva approximately every ten days, and they are obligate haematophages, taking no other food or liquid. The most pronounced isotopic differences in blood diets are the result of the fodder plant or primary producer's photosynthetic pathways, whether C3 or C4, leading to differences in carbon isotope signal of about 14‰. C3 plants are generally found in temperate climates with lower light intensity and have typical isotopic values of around -27 +/- 2‰, but range between -25‰ and -35‰ verses VPDB depending on the species and environmental conditions (O'Leary 1988). Plants such as maize, millet, sugar cane, etc. have C4 Hatch-Slack photosynthetic pathways (Hatch and Slack 1966) and thrive in sub-tropical environments. C4 plants have isotopic values of around -13.1 +/- 1.2‰ and range between -7 to -18‰ vs. VPDB with maize about -11‰ vs. VPDB (O'Leary 1988).

Differences in the isotopic signatures of nitrogen could be the result of leakiness of the nitrogen cycling in the primary producer's habitat. Intrinsic differences in isotopic signatures of the mass reared versus wild flies are extremely useful tools for population delimitation, but values can vary and may overlap (Hood-Nowotny et al. 2006, 2009). Therefore, in later stages of an elimination program more definitive tools are required, at which stage adding compounds enriched with stable isotopes to the diet of the mass reared flies is an appropriate labeling strategy because it exaggerates the isotopic difference between the wild and mass reared populations. Isotope labeling offers the opportunity to estimate vector population size and trace the distribution and movement of populations across the landscape (Wassenaar and Hobson 1998). It can also be used to determine which males, wild, sterile or genetically modified, are inseminating the native female populations, and reveals vital information about nutritional status and feeding strategies (Helinski et al. 2007; Helinski et al. 2008; Benedict et al. 2009).

The aim of this research was to demonstrate, in principle, whether the intrinsic isotopic signature of adult factory reared tsetse flies, *Glossina pallidipes* (Austen) (Diptera: Glossinidae), was sufficiently different from that of the wild populations to allow the natural abundance signatures to be used as a reliable marker to distinguish wild flies from sterile flies in the field with a high degree of confidence for the Southern Rift Valley Tsetse Eradication Project (STEP) in Ethiopia. This was achieved by determining the baseline isotope ratios of ^13/12^C, ^15/14^N ^18/16^O, and ^2/1^H of flies from the mass-rearing facility in Addis Ababa and of wild flies from the target populations in the southern rift valley in Ethiopia. It should be stressed that this was an initial proof of principle study. Isotope labeling experiments in the laboratory were also carried out using stable isotope enriched compounds to establish minimal labeling requirements for detection and turnover time of the label. This was done to estimate the potential persistence of the isotope marker during adult life post release.

The unusual life cycle of the tsetse meant that specific experiments had to be established to determine the best labeling strategy. The unusual feature of the *Glossina* life history is that the larva spends practically all its time, and does all of its feeding, within the body of the female fly. In the wild, tsetse flies probably mate near or on host animals. Females mate young and usually only once, probably around the time of taking the first blood meal. Once the egg is fertilized it remains within the female and develops into the third instar larvae when it is deposited by the female. All the larval nutrition, apart from that present in the egg yolk, of the three instars comes from the milk glands of the mother fly.

When the larva is fully grown, the female *Glossina* flies around looking for a suitable area in which to deposit it. Within an hour or two of deposition the larva pupates inside the last larval skin, which becomes barrel-shaped and darkens to form the puparium. There is no feeding by the larva after it is dropped by the female. The pupal stage usually lasts about four to five weeks. On emergence from the pupa the teneral, unfed fly will search for a blood meal and will feed about every three days. Once mated, a female can produce larvae for the rest of her life. The female fly will produce a mature larva every 9–10 days, except for the first one that takes 18–20 days from the time of emergence of the fly from the puparium.

## Materials and Methods

Mass reared *G. pallidipes* were collected from the colony reared in Kaliti, Addis Ababa (FAO/IAEA 2006). Wild *G. pallidipes* were collected in NGU-2G traps (Brightwell et al. 1991) from three routine monitoring sites in the STEP area: Dilla on the east side of lake Abaya (06.38210N, 038.05858E, 1199 MASL), Walaita Sodo on the west side (06.550881N, 37.5379944E, 1247 MASL), and Nech Sar, National Park, south of Lake Abaya (5.983494444N 37.56628889E, Alt 1172m). Sampling sites were chosen from those that were currently being surveyed for tsetse populations and that may act as future target sites for SIT.

Mass reared flies in Ethiopia and Austria were reared according to standard operational procedures developed at IAEA Laboratories Seibersdorf Briefly, adult flies were held in 20 cm diameter round mesh cages at 24° C, 70–80% relative humidity, 12:12 L:D 12–14 lux light regime, and fed sterile bovine blood through a silicone membrane three times a week, namely Mondays, Wednesdays, and Fridays. Pupal incubation temperatures were set at 24° C, 75–80% relative humidity.

### Laboratory experiment

In the laboratory isotope labeling experiment, three blood feeding treatments were set up ^15^N and ^13^C labeled blood (Table I) and an unlabelled blood control. The label was dissolved in 1ml of sterile water and added to one litre of blood which was then well mixed and poured into 50 ml aliquots, stored frozen, and defrosted prior to colony feeding. In the control only water was added, in the ^13^C treatment 0.2g of 99 atom % ^13^C labeled glucose was added per litre, and in the ^15^N treatment 0.04g of 99 atom % ^15^N labeled glycine was added per litre. Newly emerged unfed flies were taken from the colony for the experiment (parental generation, Go). Seven cages (three ^15^N, three ^13^C, and one unlabelled control) each containing 30 females and 6 males were blood fed three times a week with blood of the respective treatment. This was continued for 49 days at which point the blood was changed to unlabelled blood. The flies were maintained under standard rearing conditions (Feldmann 1994; Gooding et al. 1997; FAO/IAEA 2006). Under these conditions the G_0_ females started producing larvae about day 18–20 and produce one larva each 10 days. Pupae from the respective treatments (first offspring generation, G_1_) were collected daily and amalgamated into a single appropriate treatment emergence cage. Emerged G_1_ adult males and females were collected daily and amalgamated over a one week period into a separate rearing cage for each treatment. These were held unfed till the Monday of each week, when blood feeding with unlabelled blood commenced and was continued three times per week, giving cages of G_1_ flies with a known emergence date and the same number of feeds. The initial lack of blood feeding resulted in some mortality, but ensured sufficient replicate numbers of adult flies and the same number of feeding events. Both are an essential component of this type of study.

**Table 1.**
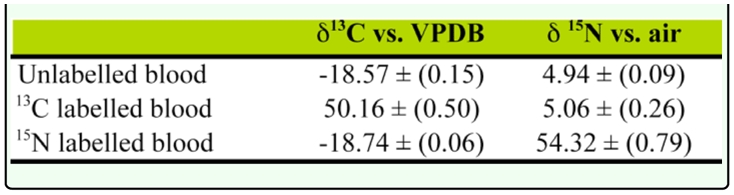
Mean isotopic values of the different blood fed to the tsetse flies. Values in parenthesis are one standard deviation of the mean value.

The G_1_ pupae used for this experiment were collected around days 45–50, whilst the Go were still being fed label, and the adults emerged between day 75 and 81. The G_1_ flies were sampled 0, 7, 14, 28, 56, and 84 days after their initial feed. Zero day flies were teneral, i.e. unfed emerged adults. A target of five replicate flies per treatment and sex was established, this was reduced to 3 replicates where insufficient flies were available.

### Sample Preparation

Flies were either individually dried (60° C for 24hrs) and stored in Eppendorf tubes with silica gel or stored in plastic tubes in the freezer and subsequently dried (laboratory experiment). Either the same leg or wing was removed from each fly or the whole body was analyzed. For ^15^N and ^13^C analysis, these were then sealed into 8mm × 5mm tin cups and analyzed using a Carlo Erba (www.carloerbareagenti.com) carbon nitrogen (CN) analyzer, linked to an Optima automated isotope ratio mass spectrometer (IRMS) (GV Instruments, www.gvinstruments.co.uk). Samples were combusted in an atmosphere of oxygen at 1700° C, and the resultant gas carried in a stream of helium through a series of scrubbers to remove sulphurous impurities and residual water, as well as over hot copper to reduce oxides of nitrogen to elemental nitrogen (N_2_). Carbon dioxide (CO_2_) and N_2_ peaks were separated on a 3m Porapak Q gas chromatography column. The CO_2_ and N_2_ peaks were then bled into the mass spectrometer to determine the isotopic ratio. For ^2^H and ^18^O analysis, the body parts were sealed in 3.5mm × 5.0 mm silver capsules and analyzed at the stable isotope facility SILVER Laboratory, University of Vienna on an isotope ratio mass spectrometer (Delta Advantage V, Thermo Fisher, www.thermoflsher.com), interface (ConFlo III, Thermo Fisher), and high temperature pyrolysis analyzer (TCEA, Thermo Fisher). Briefly samples were combusted in a glassy carbon reactor at 1400° C to CO and H_2_, and separated on a chromatographic column. Oxygen was analyzed as CO, and D/H was analyzed as H_2_.

The isotope ratios were expressed as parts per thousand (‰ or delta (δ) units) deviation from the internationally recognized standards Vienna Pee Dee Belemnite (VPDB) (Gröning 2004), atmospheric nitrogen, and Vienna Standard Mean Ocean Water (VSMOW) for carbon, nitrogen, and the oxygen and hydrogen isotopes of water, respectively.

The results were analyzed by ANOVA and Kruskall-Wallis Tests. Pair wise comparisons were made using Duncan's multiple range tests (Statgraphics, www.statgraphics.com) and where the variances were heteroscedastic the t'-test.

## Results and Discussion

**Figure 1.**
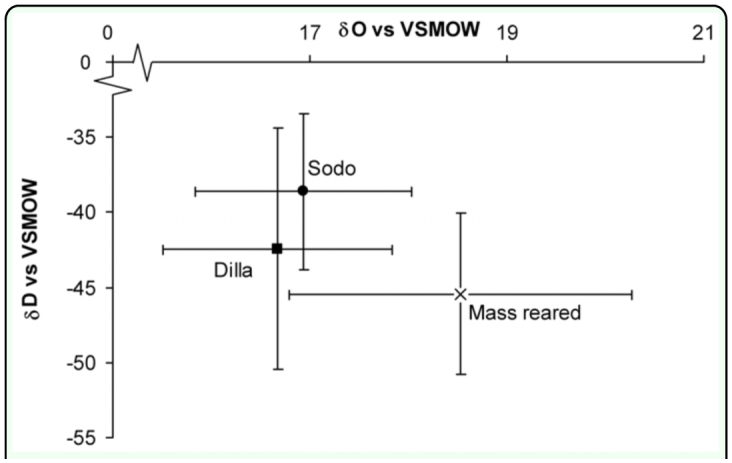
Average isotopic signature of oxygen and hydrogen in dried tsetse fly, *Glossina pallidipes* wings collected from the mass reared colony and from two locations in the wild, Sodo and Dilla, Ethiopia. Bars are plus and minus one standard deviation of the mean, n = 5. VSMOW is Vienna Standard Mean Ocean Water, an internationally recognized standard for ^2^H. High quality figures are available online

The δ^2^H (deuterium) values were highly variable with high standard deviations (Figure 1). This may reflect true differences in isotopic signature or it could be due to differences in flight activity, which can lead to excessive water loss and thus isotope discrimination or isotopic differences in source waters. For example, a small ephemeral water hole would become far more deuterium enriched than a large lake.

Colony and wild flies were not distinguishable using the δ^18^O and δ^2^H signatures. Of the isotope values of the three populations mass reared, Sodo and Dilla were not significantly different based on δ^18^O (F = 2.71, d.f = 2, 12 P = 0.1070) and δ^2^H (F = 1.46, d.f. = 2, 12 P = 0.27) data as determined using ANOVA. Flies from Nech Sar were not analysed as the preliminary data suggested it would not be a useful method. In a preliminary analysis to evaluate the optimal appendage for analysis, it was found that there was significant correlation between the ^18^O values of the legs and wings (R^2^ = 0.577 df = 9, 10 p = < 0.05) suggesting that the values are controlled by the same variables, most likely either water or food source.

**Figure 2.**
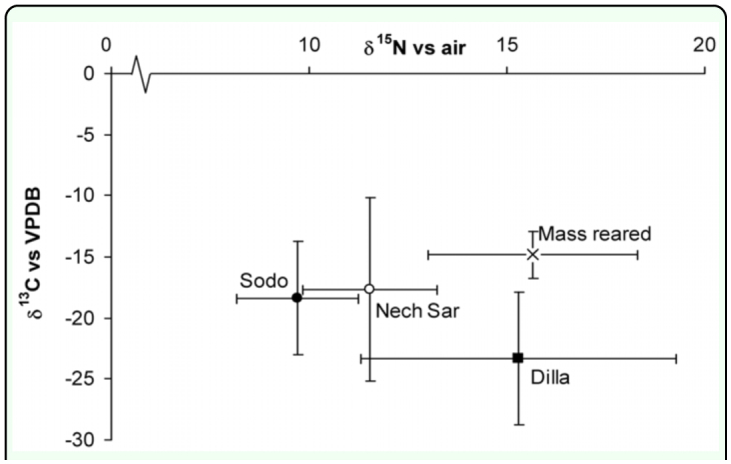
Average isotope values of tsetse flies, *Glossina pallidipes* collected from the mass rearing factory, Sodo, Dilla, or Nech Sar in Ethiopia. Error bars are two standard deviations of the mean. VPDB is Vienna Pee Dee Belemnite an internationally recognised standard for ^13^C. High quality figures are available online

The isotopic signatures of the carbon and nitrogen of the wild (Sodo, Dilla, and Nech Sar) populations were statistically significantly different from the isotopic signature of mass reared populations (ANOVA, Duncan's MRT). Although ^13^C values and contrasts showed that the wild populations were significantly different from the mass reared population (F = 17, 36, df = 3, 41 P < 0.000), it is only possible to use this method in a predictive manner to discriminate the populations of Sodo and Dilla from the mass reared population with 95% confidence as determined by separation by two standard deviations from the population mean. It was not possible to distinguish the Nech Sar population from the mass reared population (Figure 2). For prediction, reliable separation of populations is usually determined as two times the standard deviation, giving a 95% percent confidence that the populations are distinct; and this method is akin to the detection limit method in analytical chemistry (MacDougall and Crummett, 1980). The high variability in the ^13^C value of the Nech Sar population may have reflected the possible diverse wild host range of both browsers and grazers in the national park (MacDougall et al. 1980). The mass-reared population had a δ^13^C value typical of a C4 plant based diet suggesting that the blood on which the flies fed was derived from cows eating maize or another C4 plant source, which are characteristic of tropical climates. C4 plants have the Hatch-Slack photosynthetic pathway that is a common adaptation of tropical plants, resulting in a distinctly different isotopic signature (-7 to -18‰ vs. VPDB) to the more temperate C3 plants (-25‰ and -35‰ vs. VPDB) (O'Leary 1988). Sodo and Dilla populations had statistically different δ^13^C values, and this finding suggests they were feeding off hosts with significantly different plant diets. It appears that Dilla populations are feeding off a host which has a predominantly C3 diet indicating an area with a more temperate (highland) climate (the mean annual temperature of Dilla ranges between 17° C and 22.4° C, with average annual rainfall of 1800 mm (Kippie 2002)), whilst the Sodo population has a signature which suggests that the host is feeding on a C4 diet which is characteristic of more tropical (lowland) climates (the mean annual temperature of Sodo ranges between 11.9° C and 26.2° C, with average annual rainfall of 1100 mm (Kippie 2002)), or a maize residue based diet which is prevalent in the region (Tolera and Said 1992). The mass reared flies had signatures very close to the C4 diet value; as diet cow blood is collected from a commercial abattoir and it can be assumed that the cattle are being fed predominantly on a maize-based diet reflecting common agricultural practice of maize-based concentrate feeding.

^15^N values and contrasts showed that the wild populations were significantly different from the mass reared population (F = 50, 36 df = 3, 41 P < 0.000). However again it was only possible to use this method in a predictive manner to reliably discriminate the populations of Sodo and Nech Sar from the mass reared population with 95% confidence.

Interestingly, from an ecological perspective, there was a highly significant correlation (R^2^ = 0.426, P < 0.001, which increased to R^2^ = 0.694, P < 0.000001 if one possible outlier was removed from the analysis) between the δ^13^C value and the δ^15^N of the wild Sodo and Dilla flies' legs which may be attributable to the nitrogen status of the C4 and C3 primary producing plants, or which could be linked to the rainfall status leading to variation in the plant nutrient cycling in the two sites. When the Nech Sar data was included in the regression this correlation was lost, suggesting that other factors were influencing this link, such as the diverse host range in the national park. The results suggest that there is a strong link between the C3, C4 plant ecology status and the nitrogen cycling efficiency of the ecosystem, and that these ecological differences revealed by the isotopic signature are being conserved in the tertiary consumer. Enriched ^15^N values of forest foliage are indicative of a leaky N cycle (Davidson et al. 2007). The C3 plants will be dominant in the wetter nitrogen leakier environments, where nitrate leaching and gaseous nitrogen losses will be higher leading to more enriched ^15^N values and the C4 in the drier N conservative environments giving rise to the strong correlation between the ^13^C value and the ^15^N value. Another explanation would be that the higher protein content of the C3 diet, with a lower C:N ratio than the C4 plants, leads to a higher enrichment of ^15^N in the flies as it has been shown that higher levels of protein in the diet can lead to isotopic enrichments in the region of 9 per thousand (Sponheimer et al. 2003).

Although the wild populations were distinguishable from the mass reared populations using either ^15^N or ^13^C signals the results showed that there was significant inter-site variability in the isotopic signature of the wild flies. This means that for each site studied, it would be prudent to undertake an initial survey to determine whether there is isotopic separation from the mass reared population. Another issue is that the blood from the abattoir could also have a variable signal, due to different diets of the cattle reared for slaughter. Thus, it is also necessary to continually monitor the isotope ratios of the mass reared population if this technique were to be adopted. One way of overcoming these problems and increasing the certainty of the identification would be to incorporate an isotopic label to the diet of the mass reared flies. This was the rationale behind the second set of experiments.

In the second set of experiments the first weekly sample was taken on day 75. The unlabelled blood the Go flies were fed was 18.57 and 4.94 ‰ ^13^C and ^15^N, respectively; the ^13^C labeled blood was 50.16 and 5.06 ‰ ^13^C and ^15^N, respectively; and the ^15^N labeled blood was -18.74 and 54.32 ‰ ^13^C and ^15^N, respectively (Table 1). The blood was sufficiently labeled for the experiment. The control flies had a ^13^C value of around -20‰ and a ^15^N value of around 15.8 ‰. The ^15^N value of the control was higher than expected, as usually they will be close to the isotopic signature of the diet, not ten per thousand higher, suggesting some cross contamination from the ^15^N labeled blood which could have occurred by mistakenly placing the control cages on the ^15^N labeled blood.

**Figure 3.**
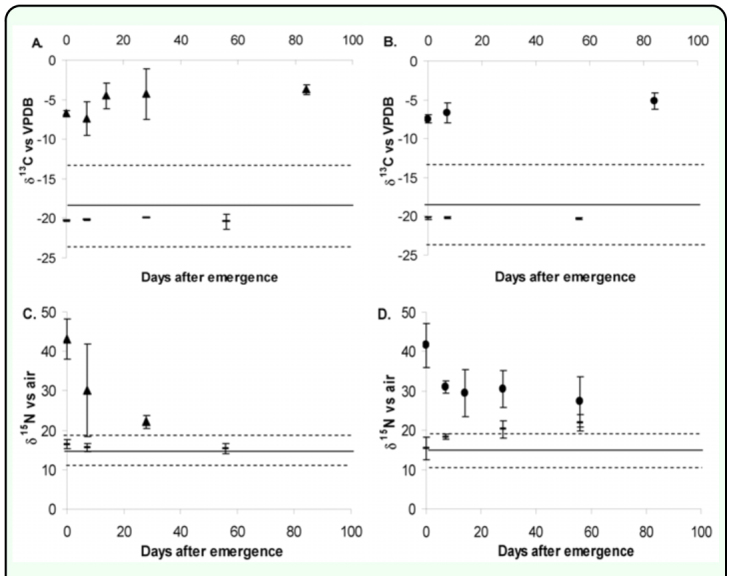
Average isotope values of laboratory reared labelled G_1_ tsetse flies *Glossina pallidipes* (^13^C triangles, ^15^N filled circles) and control un-labelled flies (short bar) at various times after emergence, error bars plus and minus two standard deviations (n = 3–5). The solid line represents the mean isotopic value of the wild population and the dashed lines, plus and minus two standard deviations of that mean value. A and C are females; B and D are males. High quality figures are available online

The ^13^C labeled male and female offspring (G_1_) were sufficiently labeled, with up to around -4‰ vs. VPDB, which was sufficiently greater than any level of isotope enrichment observed until now - in the tsetse fly environment in the field, in the controls in the laboratory (Figure 3a, b), and is higher than any values observed in terrestrial higher plants (O'Leary 1988). The δ^13^C values, however, were much lower than that of the value of the blood that the adult flies were fed (50 versus -4‰), suggesting that a high proportion of initial body carbon (probably fat) is used for subsequent larval nutrition or that there was a degree of dilution from unlabelled blood sources suggesting high carbon turnover rates. There was no apparent decline in the ^13^C of flies emerged from the labelled adults over time in this batch of samples, which could suggest that the ^13^C label that was present was incorporated in to tissues which were not metabolically active and that ^13^C levels had reached equilibrium levels. The slight increase in the ^13^C value over time could suggest some sampling error, or it could be attributed to the natural variability in blood uptake of the tsetse population. Despite this variability, the results indicate that this method of marking tsetse flies with ^13^C labeled glucose is a technically viable marking method for tsetse flies which would enable clear delimitation of wild populations from mass reared populations over 80 days - a time span greater than the expected life span of an irradiated, mass reared fly.

**Figure 4.**
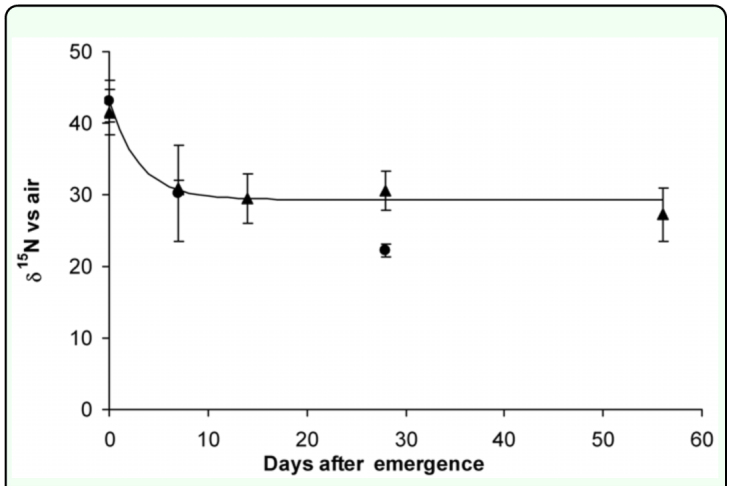
Decline in ^15^N enrichment of tsetse flies, *Glossina pallidipes.* Line is modelled decline in the ^15^N value of females (y = 13.741 e(^-0.3322x^) + 29.3). Circles are observed male values; triangles observed female values. High quality figures are available online

The ^15^N labeled female and male flies were sufficiently labeled (greater than two standard deviations) above the δ^15^N values observed in the field which would allow for identification of mass reared flies (Figure 3c, d). However there was some overlap between the experimental control and the ^15^N labeled flies at the latter end of the experiment in the female samples as the δ^15^N of control flies increased significantly during the experiment, possibly due to protein turnover associated with pupal production in addition to some ambiguity over the initial control values. As only male flies are released and the isotope values obtained were sufficiently above those of the observed wild populations, it can be concluded that labeling was sufficient to categorically distinguish wild from mass reared populations even after several weeks of unlabelled feeding. The teneral value of the newly emerged tsetse was around 42 ‰ 15N, which was only about ten per thousand less than the value of the blood fed to the parents. This suggests that the adult blood feeding provides up to 80% of the N requirement (probably protein) for larval development, and that only a small fraction of existing adult body N is used to provide nutrients for larval development; this explains why tsetse that do not blood feed frequently enough do not produce viable larvae or fail to produce larvae (Gaston and Randolph 1993). Interestingly, teneral flies from labeled parents showed a rapid characteristic curvilinear decline in the isotopic value for nitrogen when switched to an unlabelled N source (Haq et al. 2010), which suggests rapid turnover of protein in the tsetse fly when fed blood, with a half life of about 7 days as it took only 14 days to reach full isotopic equilibrium. There were almost no differences between female and male N turnover rates as determined by observation of the ^15^N values, an observation which is in concurrence with the findings of Foster (1976) who stressed the importance of blood feeding in males in relation to sexual performance. A model based on simple exponential decay (Figure 4) appeared to confirm that there was little difference in the male and female N assimilation values. The model was constructed from the initial measured values using simple equations; these were then run on a daily time step. Goodness of fit of the model was assessed, calculating the square of the deviation of the observed from modeled values. The model with the least sum of squares was deemed the best fit that in both cases seemed to be that based on the female data. An attempt was made to model the data based on an exponential decay from the initial male data, but this model did not fit the male data as well as the female turnover model.

The modeled data and observed data suggested a curvilinear decline in the ^15^N value, which can be attributed to the turnover of N in the fly — initially the ^15^N value declines exponentially as the metabolically active labeled N is replaced by unlabelled N as the flies feed on unlabelled blood. When the entire metabolically active N has been replaced by unlabelled N there is linearization of the ^15^N value, and it is possible to calculate the amount of metabolically inert, or structural, N in the fly using a two pool mixing model (Haq et al. 2010).

In females 64% of structural N was retained, whereas in males only 46% was retained. This is probably explained by physiological differences between the male and female flies. These values are similar to carbon values reported for male mosquitoes and fruit flies in which around 50% of the body mass is structural (Hood-Nowotny et al. 2006; 2009). The fact that the label is conserved in the structural tissue ensures that if the flies are sufficiently labeled they will maintain a measurable ^15^N signature even after dying, a useful characteristic of the isotope marker.

### Conclusions

The isotope labeling with ^15^N and ^13^C was sufficient to distinguish mass reared populations from wild populations. However labeling and analysis costs are probably too high for this technique to be used in the initial stages of an elimination program, and would only be useful in the latter stages of the program where it is imperative that mass reared versus wild identifications are reliable as a backup to dye marking or other techniques. Calculations of the amount of isotope required to adequately label the mass reared flies suggested that ^15^N labeling would be a more financially feasible alternative to ^13^C labeling with a cost in the region of 0.004 US$ per fly released for ^15^N labeling and 0.02 US$ for ^13^C labeling, which is approximately 5% and 28% of the current estimated production costs, respectively. These costs do not account for the isotope analysis costs which are currently in the region of 5 US$ per sample using isotope ratio mass spectrometry, however new field-robust laser systems for such carbon isotope analysis have recently become available which could bring the analysis costs down to around 0.5 US$ per sample. Thus the preferred marking method becomes a strategic program management decision, but these data show that it is a feasible and reliable method.

Natural differences in isotopic signatures of mass reared and wild tsetse flies were sufficient to distinguish populations with 95% certainty, and therefore natural isotope signatures could be useful complimentary identification markers in cases of dye marking uncertainty. Isotope labeling proved to be a simple and economically viable means of labeling a tsetse fly population that could easily be integrated in to a tsetse fly SIT rearing program.

## Abbreviations

**AW-IPM**,: Areawide integrated pest management;
**SIT**,: Sterile insect technique;
**VPDB**,: Vienna Pee Dee Belemnite;
**VSMOW**,: Vienna Standard Mean Ocean Water
